# Melanoma genome evolution across species

**DOI:** 10.1186/s12864-017-3518-8

**Published:** 2017-02-07

**Authors:** Emily R. Kansler, Akanksha Verma, Erin M. Langdon, Theresa Simon-Vermot, Alexandra Yin, William Lee, Marc Attiyeh, Olivier Elemento, Richard M. White

**Affiliations:** 1 Memorial Sloan Kettering Cancer Center, Cancer Biology & Genetics, New York, USA; 2 Weill-Cornell Medical College, Institute for Computational Biomedicine, New York, USA; 3 Memorial Sloan Kettering Cancer Center, Computational Biology, New York, USA; 4 Memorial Sloan Kettering Cancer Center, The David M. Rubenstein Center for Pancreatic Cancer Research, New York, USA; 5 Department of Medicine, Memorial Sloan Kettering Cancer Center, New York, USA

**Keywords:** Cancer, Zebrafish, Drug-resistance, Melanoma

## Abstract

**Background:**

Cancer genomes evolve in both space and time, which contributes to the genetic heterogeneity that underlies tumor progression and drug resistance. In human melanoma, identifying mechanistically important events in tumor evolution is hampered due to the high background mutation rate from ultraviolet (UV) light. Cross-species oncogenomics is a powerful tool for identifying these core events, in which transgenically well-defined animal models of cancer are compared to human cancers to identify key conserved alterations.

**Results:**

We use a zebrafish model of tumor progression and drug resistance for cross-species genomic analysis in melanoma. Zebrafish transgenic tumors are initiated with just 2 genetic lesions, BRAF^V600E^ and p53^-/-^, yet take 4–6 months to appear, at which time whole genome sequencing demonstrated >3,000 new mutations. An additional 4-month exposure to the BRAF inhibitor vemurafenib resulted in a highly drug resistant tumor that showed 3 additional new DNA mutations in the genes BUB1B, PINK1, and COL16A1. These genetic changes in drug resistance are accompanied by a massive reorganization of the transcriptome, with differential RNA expression of over 800 genes, centered on alterations in cAMP and PKA signaling. By comparing both the DNA and mRNA changes to a large panel of human melanomas, we find that there is a highly significant enrichment of these alterations in human patients with vemurafenib resistant disease.

**Conclusions:**

Our results suggest that targeting of alterations that are conserved between zebrafish and humans may offer new avenues for therapeutic intervention. The approaches described here will be broadly applicable to the diverse array of cancer models available in the zebrafish, which can be used to inform human cancer genomics.

**Electronic supplementary material:**

The online version of this article (doi:10.1186/s12864-017-3518-8) contains supplementary material, which is available to authorized users.

## Background

Large-scale advances in genomic profiling of human cancers has enabled the identification of thousands of new potential genetic and epigenetic targets [1]. In melanoma, this effort has been complicated by the relatively high background mutation rate induced by ultraviolet light (UV) [2]. This is further complicated by recent observations that even seemingly normal skin harbors large mutational burdens due to UV, [3], making it difficult to discern which events are truly pathogenic versus those that occur simply as bystanders.

Along with ultraviolet light, human melanomas also exhibit tremendous inter-patient heterogeneity. This is likely due to several factors: 1) a variety of genes can act as initiating events (i.e. BRAF, NRAS, c-Kit), 2) a lack of knowledge of the cell of origin of individual tumors, and 3) individual germline variation in DNA repair mechanisms. As the tumors evolve under drug therapy (i.e. BRAF inhibitors or immunotherapy), each of these factors make it increasingly challenging to identify key genomic events promoting such evolution.

Evaluating genetic evolution in melanoma will be facilitated by models which faithfully recapitulate the human disease, yet allow for precise control over the above variables. This will allow for identification of core mutational events and mechanisms that are intrinsic to melanoma, and not simply due to the background effects of UV radiation. Towards this end, we and others have previously developed several zebrafish models of melanoma [4–7] that show remarkable similarity to the human disease at histological, functional and genomic levels [8]. The zebrafish has emerged as an important new model in cancer biology because of its unique capabilities in terms of transgenesis, genetic manipulation, unbiased screens and in vivo imaging [9]. The advantages of evaluating cancer evolution in models such as the fish is that we can rigorously control the cell of tumor initiation, use genetically well-defined oncogenic initiating events and the fish spontaneously develop melanoma in a well-defined germline background.

One limitation of using these transgenic models of cancer is a lack of computational methods for assessing the tumor genomes over time and space. We previously utilized an exome-sequencing approach to identify genes under selection in these melanomas [8], but did not address mutation rates genome-wide or how this changes during the emergence of drug resistance. Such an approach will be necessary to truly capitalize on the strengths of the zebrafish cancer models.

**Fig. 1:**
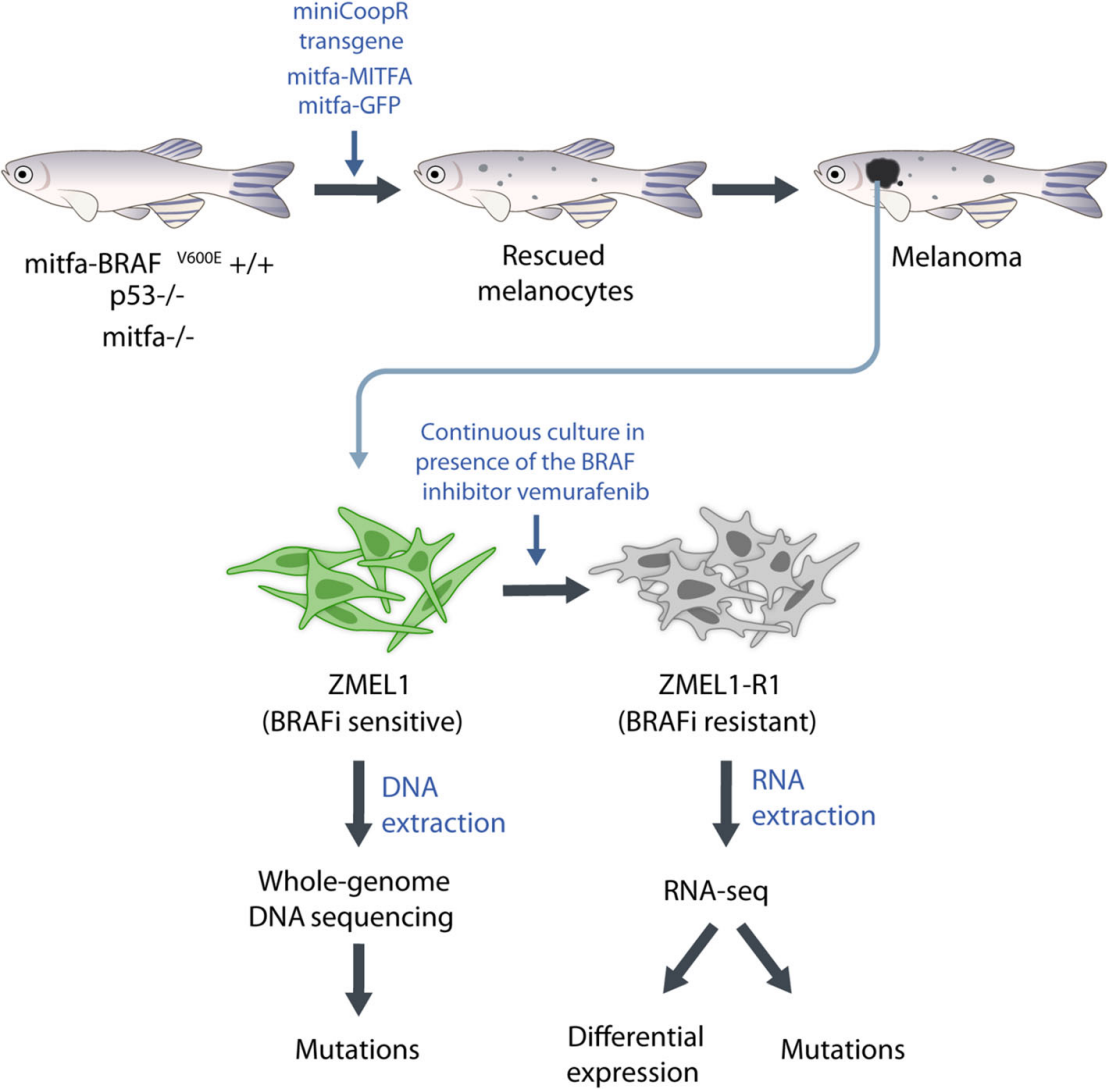
Schematic representation of experimental plan. Fish with the genotype mitfa-BRAFV600E+/+;p53-/-;mitfa-/- are completely devoid of melanocytes due to a mutation in the mitfa gene, but carry the BRAFV600E transgene in their germline. Upon transgenic expression of a miniCoopR rescue cassette in which the mitfa promoter drives an MITFA minigene, the animals develop mosaic resuce of melanocytes. A small number of these melanocytes will eventually complete all the steps of malignant transformation, and emerge as a cutaneous melanoma. From one such melanoma, a stable cell line, ZMEL1, was derived. Genomic DNA was isolated from this line, along with normal DNA from the original fish from which the tumor was derived. These DNA samples were used for whole-genome sequencing to call mutations specific to the ZMEL1 tumor. The ZMEL1 line is highly sensitive to growth inhibition by BRAF inhibitors, as expected. Continuous culture for of the ZMEL1 line in the presence of high dose of the BRAF inhibitor vemurafenib for 4 months gave rise to a derivative cell line, ZMEL-R1, which demonstrates a 10-fold reduction in sensitivity to vemurafenib. RNA was isolated from this line, along with RNA from the parental ZMEL1 line, which was then used for both differential expression analysis as well as mutation calling

Here, we utilize a transgenic zebrafish melanoma to define genetic events that occur in the months after the initiation of BRAF^V600E^ expression, and determine how these tumors evolve under drug selection with a BRAF inhibitor, as schematically demonstrated in Fig. 1. These data reveal a surprising amount of genetic evolution that occurs in the absence of UV damage, which can be further augmented after these melanomas become resistant to BRAF inhibitors. These data suggest that the mechanisms leading to genomic instability in melanoma may not solely be due to a straightforward mutagenic insult such as UV, and likely reflects properties of the melanoma cell itself.

We use these new approaches to compare the zebrafish genomes to human melanoma genomes. During drug resistance, we find strong conservation of both DNA mutations as well as mRNA transcriptional profiles. This data highlights the capacity for cross-species oncogenomic approaches to filter out the highly noisy changes seen in human melanoma, and identify core mechanisms of tumor progression and drug resistance. These methods can be broadly applied to other tumor types.

## Results

### ZMEL1 line derivation

For these studies, we utilized the previously developed zebrafish melanoma model [6, 7], in which human oncogenic BRAF^V600E^ is expressed under the melanocyte specific mitfa promoter. We generated these melanomas using the mosaic miniCoopR system [10, 11]. The stable transgenic mitfa-BRAF^V600E^ animals were crossed into animals with homozygous mutations in p53 and mitfa itself. These animals are completely devoid of melanocytes due to the mitfa mutation [12], but harbor the capacity for melanoma initiation when mosaically injected with a transgene containing an mitfa and GFP rescue cassette. From one such animal at 6 months of age, we extracted a large GFP+ melanoma from the skin, and then established a stable GFP+ zebrafish melanoma cell line called ZMEL1 (Fig. 1), as previously described [13]. This line was used for all subsequent studies.

### Whole-genome sequencing of the ZMEL1 melanoma line

Since the tumor from which the ZMEL1 line was initiated with only 2 genetic lesions, BRAF^V600E^ activation and p53 loss of function, characterization of its genome gave us an opportunity to characterize further genetic evolution that contributed to tumor formation over these 6 months. We extracted genomic DNA from this stable cell line, along with normal genomic DNA from the muscle of the original transgenic animal. These 2 samples were then subject to Illumina sequencing to a depth of 39X for both the tumor and normal DNA.

### Point mutations in ZMEL1

We aligned the Illumina reads using the GSNAP aligner [14], which is SNP tolerant and we felt would perform better than BWA given the generally high levels of SNPs seen in the zebrafish genome compared to the human genome. Because most mutation calling pipelines are optimized for the human genome,we decided to run two separate pipelines, MuTect [15] and Shimmer [16], in order to optimize the balance between false-positives and false-negatives.

**Fig. 2:**
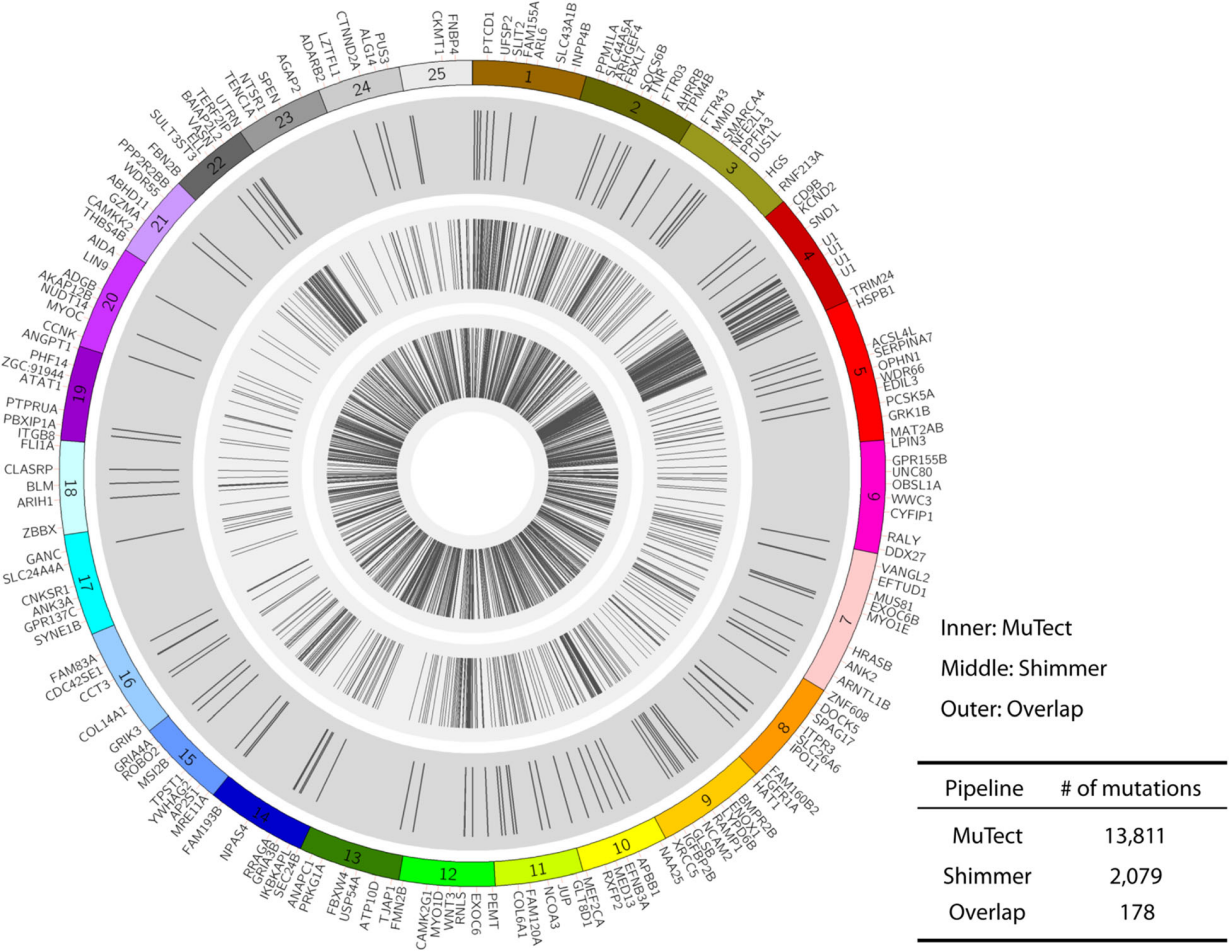
Circos plot showing called mutations in the ZMEL1 line. MuTect called the greatest number of mutations, as shown in the inner ring, with far fewer called by Shimmer (middle ring). The number of overlapping mutations, which have a higher validation rate, is shown in the outermost ring of the plot

As seen in Fig. 2 and Additional file 1: Table S1, the MuTect algorithm called a total of 13,811 potential point mutations (Fig. 2, inner ring) in the ZMEL1 tumor, whereas Shimmer called a much smaller number at 2079 mutations (Fig. 2, middle ring). Looking at the overlap between these two mutation callers, we surprisingly found only 178 mutations (Fig. 2, outer ring) in common. This is a higher degree of non-concordance than is typically seen in human cancer samples, but likely reflects the fact that the zebrafish genome is more repetitive than the human genome, with a much higher frequency of SNPs [17], such that a sequencing depth of 39X may not be fully sufficient for the mutation calling algorithms to perform optimally.

**Fig. 3:**
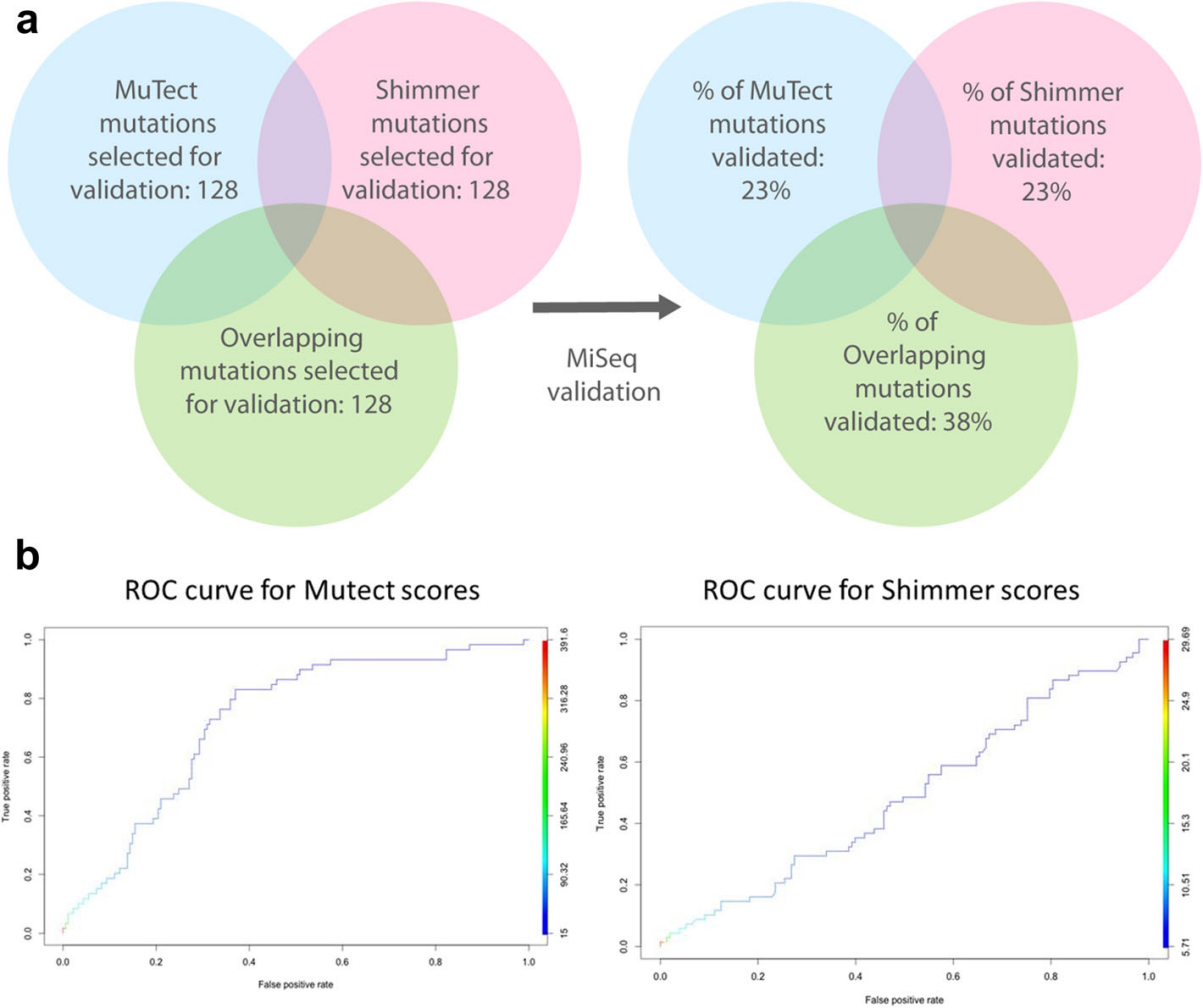
**a** MiSeq validation of a subset of ZMEL1 mutations. A total of 384 variants were chosen, including 128 unique to Mutect or Shimmer and 128 that overlapped between the two callers. Whereas each pipeline individually only showed a 23% validation rate, this could be significantly increased to 38% by looking at the overlapping mutation calls. **b** Receiver-operating characteristic curves for MuTect and Shimmer show that higher MuTect scores are associated with a greater likelihood of a verified mutation, whereas the Shimmer quality scores have little relationship to validation outcome

To further determine the performance of each mutation caller, we validated a subset of the called variants from each pipeline (Fig. 3a). For each caller, we stratified the mutations into quartiles based on quality scores, and then selected representative mutations from each quartile for validation. In total, we selected 384 loci for validation (Additional file 2: Table S2 and Additional file 3: Table S3), including 128 mutations that were called by both callers, and 128 mutations uniquely called by either MuTect, and 128 uniquely called by Shimmer (Fig. 3, left). We PCR amplified each genomic locus from the originally isolated genomic DNA, and then used a pooled MiSeq run to assess whether the called mutations from the HiSeq run were correct. Overall, both MuTect and Shimmer produced a validation rate of 23%, with 60/256 for MuTect and 59/256 for Shimmer. However, the overlapping mutations in MuTect and Shimmer produced a significantly higher validation rate of 38% (49 out of 128 mutations called by both pipelines, Fig. 3, right)). The majority of the false positives we found in the validation run were due to germline SNPs that were not detected in the initial low-coverage HiSeq run, suggesting that greater sequencing depth would improve these results further.

We also wished to determine whether the quality scores produced by each mutation calling pipeline could serve as a guide to weeding out false positives. To assess this, we constructed ROC (Receiver Operating Characteristic) curves and then analyzed the sensitivity of each caller separately or together. As seen in Fig. 3b, MuTect generally performed better by ROC analysis, with an estimation that the higher quality scores were more likely to validate in the MiSeq run. In contrast, Shimmer quality scores generally had little relationship to actual validation status on the MiSeq run.

Overall, we conclude that for mutation calling from zebrafish tumor DNA, using multiple mutational callers and identifying the overlapping mutations, especially those with high quality MuTect scores, is most likely to yield mutations that can be validated by an orthogonal method.

### Nature of the mutations

**Fig. 4:**
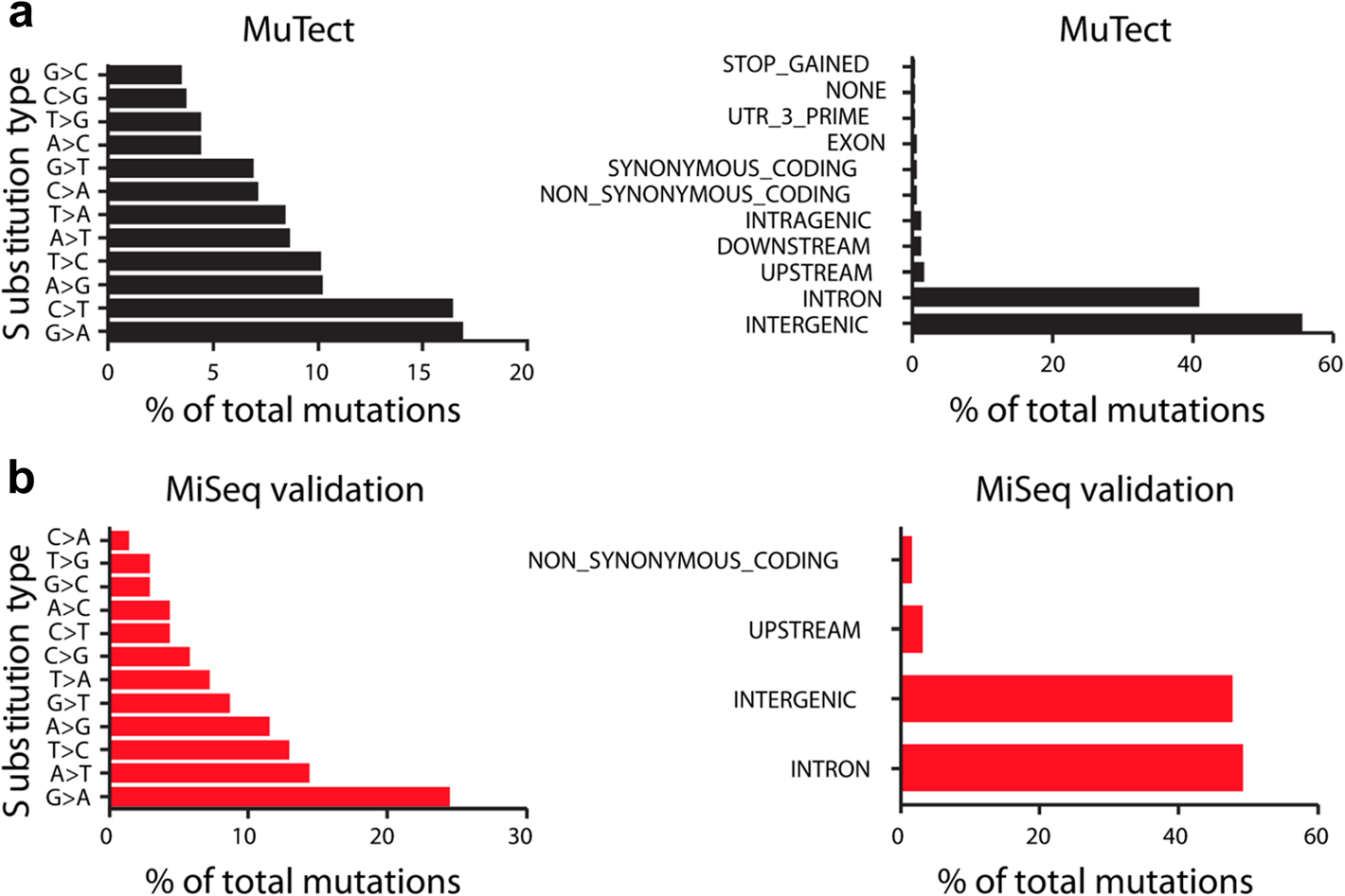
Nature of the ZMEL1 mutations. The substitution patterns and changes found for MuTect (**a**), compared to the patterns found for the mutations actually validated by the MiSeq run (**b**). In both cases, G > A substitutions, primarily in intergenic or intronic regions, predominated

Given the better overall performance of MuTect, we quantified the type of substitutions we found either before or after the MiSeq validation run (Fig. 4). As expected, the the vast majority (55–65%) of the point mutations occurred in intergenic regions, with another 28–40% occurring in intronic regions. As expected based on the exonic component of the genome, approximately 1.4–2.5% of the mutations occurred with gene bodies. Thus, there does not appear to be strong selection for mutations that occur only in exons, and instead these are spread across the genome fairly evenly. In both the raw MuTect data (Fig. 4a) as well as the MiSeq validation run (Fig. 4b), the majority of the identified mutations were G > A/C > T substitutions. These did not show a strong propensity for being dipyrimidine dimers as would be expected if these were UV-light induced: 17% of the mutations occurred at TCA triplets, and another 10% at TCG triplets, with only 5% at TCC triplets (Additional file 1: Table S1). In general, across all human cancers C > T substitutions tend to be the most common [18], consistent with our dataset.

### Comparison to human melanoma

We wanted to compare the overall mutation rates in the zebrafish tumor to human melanomas. It is likely that the 13,811 mutations called by MuTect suffered from both false positives and false negatives. If we estimate that only 23% of those mutations were true, this would yield an expected number of genome-wide mutations to be 3176 mutations, or 2.24 mutations per Mb (3176/1,412,464,843). We then compared this rough estimate to a recently published series of 25 human melanomas that had been subject to whole-genome sequencing at similar depths [19]. Strikingly, in that study, the average number of point mutations was 78,775, yielding an average mutation rate of 30 mutations per Mb. However, the number of mutations varied by two orders of magnitude in that study, ranging from 3–111 mutations per Mb. The zebrafish melanoma mutation rate was similar to that reported for acral melanomas (3–14 mutations per Mb) which are typically non-sun exposed melanomas and therefore devoid of UV mutagenesis. This data strongly suggests that the mutation rate in the zebrafish tumors reflects the near complete absence of UV exposure, and that the additional mutations that occur after initiation with BRAF;p53 are due to either clonal expansion or ongoing genomic instability.

### Derivation of the ZMELR1 cell line

We next wanted to determine how this melanoma evolved under drug selection in the emergence of resistance. In both patients and in human cancer cell lines, continued exposure to inhibitors of the MAP kinase pathway leads to eventual drug resistance in nearly all cases [20, 21]. This is true for both a variety of BRAF^V600E^ inhibitors (i.e. vemurafenib, dabrafenib) as well as MEK inhibitors (i.e. trametinib), and the mechanisms of such resistance have invariably demonstrated reactivation of the MAP kinase pathway [22].

To generate an analogous situation, we exposed the ZMEL1 cells to the BRAF inhibitor vemurafenib (1 μM) for a period of 4 months. This led to an initial die off of nearly all cells, as expected, but with a small population of persisters that were static in their growth initially but eventually completely resumed growth and re-established the culture. We call this resistant line ZMELR1, and it exhibits a nearly 10-fold increase in the IC_50_ to vemurafenib.

### Genomic analysis of the ZMELR1 cells

To understand the changes that occurred in the ZMELR1 cells, compared to the parental ZMEL1 cells, we chose to take advantage of recent advances in using RNA-seq to simultaneously analyze: 1) differentially expressed genes via analysis of the mRNA transcripts, and 2) novel mutations in the underlying DNA via recently described mutation calling pipelines using mRNA-seq [23, 24].

For this analysis, we isolated total mRNA from ~10^6^ cells from either ZMEL1 or ZMELR1 cells, and then used polyA enrichment followed by standard Illumina library preparation. We did this using three independent biological replicates. We then sequenced each sample using 100 bp PE sequencing for ~25x10^6^ reads per sample. The samples were aligned to the genome using the Tophat/Bowtie package, and then used DeSeq2 to identify differentially expressed genes. We used the mapped reads as input into the Strelka mutation calling pipeline, as previously described, in order to identify candidate point mutations.

### RNA differential expression analysis in ZMELR1 vs. ZMEL1

**Fig. 5:**
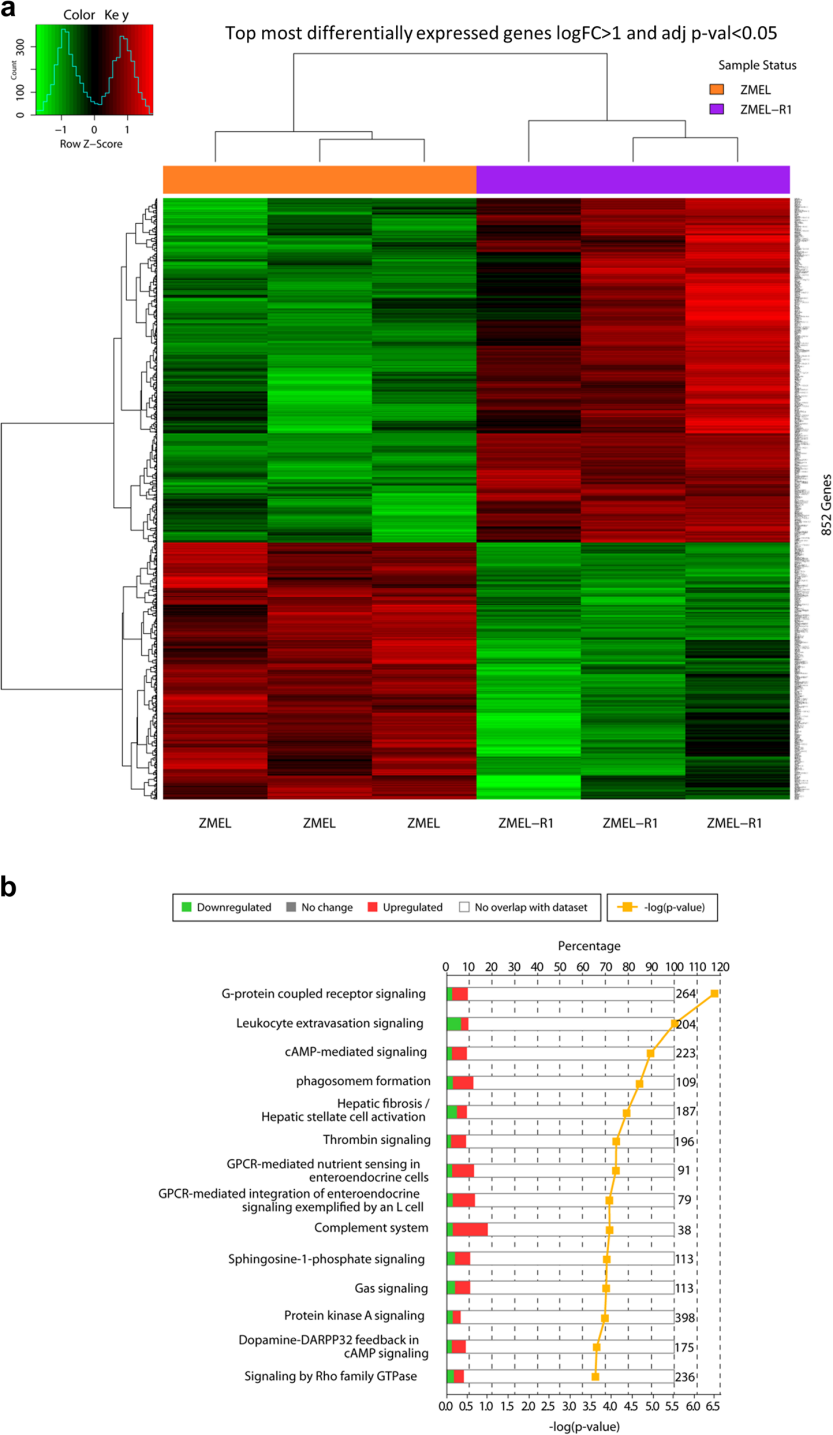
**a**. Heatmap showing mRNA expression of the ZMEL1 line versus the vemurafenib-resistant derivative line ZMEL-R1, revealing 852 differentially expressed genes. **b**. Ingenuity Pathway Analysis of the affected genes is dominated by alterations in G-protein, cAMP and PKA signaling

We identified 852 genes that were differentially expressed in the ZMELR1 cells compared to the parental cells (FDR < 0.05, FC > 2.0), including 488 that were upregulated and 364 that were downregulated (Fig. 5a and Additional file 4: Table S4). To identify the pathways that were altered, we first converted these genes into their human orthologs using the DIOPT tool (http://www.flyrnai.org/cgi-bin/DRSC_orthologs.pl) and then input these into Ingenuity Pathway Analysis. The most significantly altered pathways (Fig. 5b) included those genes involved in G-protein coupled signaling (ADCY2, ADCY5, CALCR, PDE8B), cAMP signaling (ADCY2, PDE8B, VIPR2, ADRA2C), protein kinase A signaling (ADCY2, PLCD1, PTGS2, PLC1) and leukocyte extravasation (ITGAM, PRKCB, MMP15, ITGA1).

**Fig. 6:**
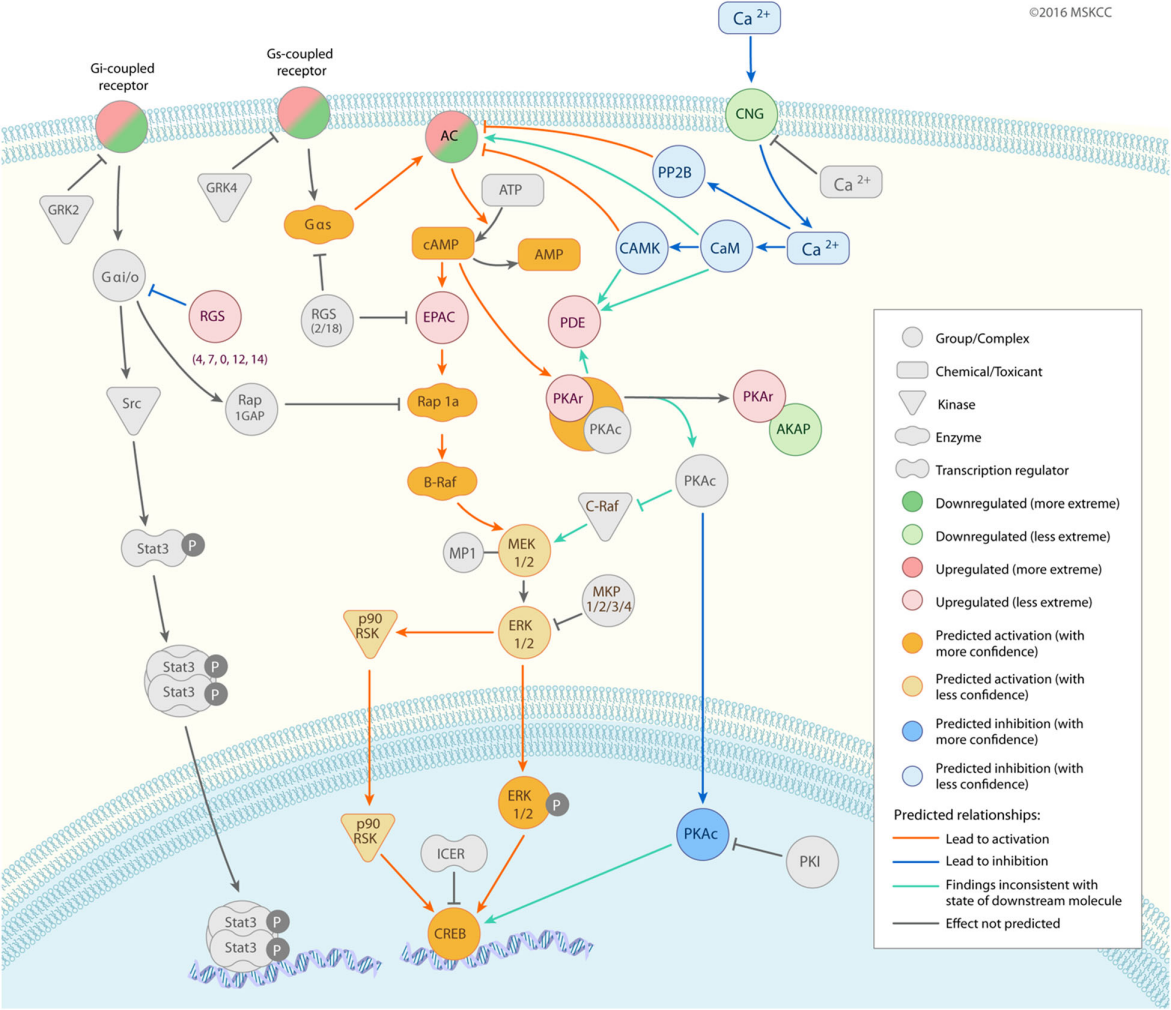
Pathway analysis of the G-protein, cAMP/PKA signaling alterations found in the ZMEL-R1 cells. This pathway diagram predicts reactivation of MAP kinase signaling through this pathway, which accounts for the resistance to the BRAF inhibitor vemurafenib

This pathway analysis is remarkably similar to what has been found in human melanomas that have become resistant to BRAF inhibitors [25]. Johannessen performed a screen in human melanoma cells that had been engineered to overexpress 15,000 open reading frames via cDNA transfection. In their analysis, G-protein coupled receptor signaling emerged as the top-ranked protein class the conferred resistance to BRAF inhibitors across a range of cell lines. The specifically identified downstream activation of adenyl cyclase, which leads to increased cAMP levels, as the critical effector of BRAF resistance. The human screen identified the adenyl cyclase gene ADCY9 and the protein kinase A subunit PRKACA as mediators of resistance, whereas we find upregulation of ADCY2 and ADCY5 and other components of PKA signaling as significantly upregulated (Fig. 6). Looking at the overlap between the upregulated genes from the ZMELR1 to those found in the Johannessen study yielded 3 additional genes that are likely components of this core resistance mechanism: SP8, NR4A2, and GPR161. These are representative of the major classes of resistance factors identified in the human screen, including G-protein coupled signaling and transcription factors. These data suggest that core mechanisms of drug resistance are tightly conserved across species. Functional validation of these conserved mechanisms awaits further study, but by directly comparing the zebrafish to human pathways and genes, we can rapidly identify new targets that may be useful in treating drug resistance in melanoma.

### DNA mutation calling in the ZMELR1 vs. ZMEL1

We next wanted to explore whether we could use the RNA-seq data to identify potential genetic changes in the underlying DNA that could contribute to BRAF inhibitor resistance. As has been well-described in human melanoma, the emergence of new point mutants often underlies clinical resistance to these drugs. These point mutations can often be identified retrospectively in the bulk population of tumor prior to therapy. We used the RNA samples from above, and after mapping applied the Strelka algorithm to identify candidate point mutations, as described previously and adapted in the methods, below [23, 24].

**Fig. 7:**
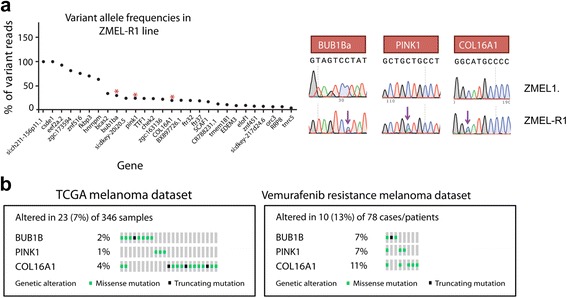
Mutations identified in the ZMELR1 vemurafenib resistant melanoma. **a**. A subset of the mutations putatively called by the RNA-seq is shown, along with their variant allele frequencies. Only 3 of the 27 tested mutations were shown to have bona-fide mutations in the underlying DNA, as demonstrated by Sanger sequencing of genomic DNA from the ZMEL1 and ZMEL-R1 lines. **b**. Analysis of these mutations in the TCGA and vemurafenib-resistant melanoma samples shows an enrichment of these in the drug resistant human tumors

Overall, we identified 51 potential point mutations in the ZMELR1 line compared to the ZMEL1 line (Additional file 5: Table S5). We reasoned that many of these were likely false positives, based on two factors: 1) the above-described suboptimal performance of mutation calling pipelines in zebrafish data, and 2) the fact that these were called from RNA rather than DNA, so may represent either allele specific expression or RNA editing events. Given these challenges, we decided to orthogonally validate a subset of these potential mutations, similar to what we did for the ZMEL1 whole-genome sequencing described above. We selected mutations with a range of variant-allele fractions, 3 to 100%. Since the mutations in this case were called in the mRNA, to validate these we isolated genomic DNA from the ZMEL1 and ZMELR1 cells, and successfully PCR amplified 28 candidate loci. These were then subject to Sanger sequencing, and the traces were manually inspected to look for the presence of mutated peaks. We found that 11.1% (3/27) of the mutations were validated (Fig. 7a): a C > T substitution in BUB1BA, a C > A substitution in COL16A1, and a C > A substitution in PINK1. Not unexpectedly, since we initially identified these mutations in the mRNA, we find that the rate of validation is lower than what we found in the whole genome DNA-sequencing of the ZMEL1 cells described above (i.e. 11% in the mRNA/Strelka pipeline vs. 38% in the DNA/MuTect + Shimmer pipeline).

We compared the mutations in these genes to those known to be mutated in human melanomas (Fig. 7b). We first looked for evidence of these mutations in all patients within the TCGA cohort of 346 patients, and found mutations in all 3 of these genes: BUB1B (2% of patients), PINK1 (1% of patients) and COL16A1 (4% of patients). We then determined whether the frequency of the mutations increased in a population that have similarly become resistant to BRAF inhibitors. Van Allen performed exome sequencing on 48 human patients who had developed clinical resistance to BRAF inhibition [26], including both pre and post treatment biopsies. Of the 78 available sequenced tumor samples, we found increases in the mutation frequency of all 3 of these genes: BUB1B increased from 2 to 7% of patients, PINK1 from 1 to 7% of patients, and COL16A1 from 4 to 11% of patients. This data suggests that core mechanisms of drug resistance are highly conserved across species, highlighting the utility of zebrafish models for identifying the mechanisms of such resistance.

## Discussion

The patterns of genetic evolution in melanoma are increasingly well-defined via large scale efforts such as The Cancer Genome Atlas. To date, over 400 patient tumors have been sequenced, primarily using whole-exome approaches [27]. A smaller number of patients have been sequenced using whole-genome approaches [19]. The majority of these cases are samples derived from cutaneous, UV-associated melanoma and as a result have very high mutation burdens. An extreme of this is desmoplastic melanoma, a histologically unique subtype occurring in chronically sun damaged skin which harbors 62 mutations/Mb [28]. These high mutation rates induced by UV complicates the analysis of true somatic evolution to a certain degree because of the very high rate of somatic mosaicism recently found to exist within normal human skin. Unexpectedly, several common cancer associated genes are found to be mutated in seemingly normal skin as well [3], calling into question the precise manner in which these mutations contribute to melanoma pathogenesis. Other types of melanomas are also being increasingly sequenced, most notably acral and mucosal melanomas [29, 30], which occur on non-sun exposed areas and have a much lower mutation rate.

Animal models such as the zebrafish and mouse are increasingly utilized to functionally dissect the mutations identified from human genomic studies such as the TCGA. For example, the zebrafish miniCoopR system, a mosaic genetic overexpression system, was used to determine which of the genes amplified on human chromosome 1q21 were likely “drivers” [11]. In that study, all of the genes amplified in that human region were overexpressed in concert with BRAF^V600E^ and it was found that only a single gene, SETDB1, contributed to melanoma pathogenesis. Aside from these functional validation models, relatively less attention has been paid to using these models to understand fundamental mechanisms of mutation accumulation and genetic evolution after tumor initiation [8].

In the current study, we aimed to understand how much further genetic evolution occurs after initiating a melanoma with just two genetic lesions: BRAF^V600E^ overexpression and p53 inactivation. We had strong reasons to think that these two lesions alone are not adequate to give rise to clinically detectable tumors. We recently showed that after initiation with BRAF and p53, the melanocytes undergo epigenetic reprogramming to a neural crest-like state via reorganization of H3K27Ac mediated-transcription [31]. However, despite the fact that BRAF and p53 are present from the time of birth, tumors rarely appear prior to 4–6 months of age. These data strongly hinted that there were further genetic and perhaps epigenetic events occurring during this tumor latency period between birth and 6 months. Since zebrafish in the lab are shielded from UV irradiation, these changes would have to occur in the absence of this known skin mutagen.

Our data supports a model in which a melanoma that is initiated with the combination of BRAF and p53 can lead to further accumulation of thousands of mutations. Many of these mutations bear the common C > T pattern seen in most cancers (non-dipyrimidine dimer types that occur independent of UV), but also other less common mutations such as the T > C/A > G pattern. When compared to a recently published compendium of mutation spectrum across human tumors, we find that the zebrafish tumors are closest, but not exactly so, to mutational signature 21 [18], which is poorly understood and likely due to as yet unexplained factors in DNA repair.

In human patients with melanoma, the genetic heterogeneity present at baseline can make the identification of drug resistance mutations difficult. The zebrafish offers an advantage in this regard, since we are starting with a relatively clean genetic background. This can allow us to rapidly filter down all of the potential new resistance mechanisms to those conserved across millions of years of vertebrate evolution. In the zebrafish melanoma, we found relatively little genetic evolution occurs after the establishment of drug resistance, with only 3 new mutations identified. In contrast, we found a very significant alteration in gene expression, with over 800 genes differentially expressed between the parental versus drug resistant tumor. It is possible that the gene expression changes we see are either directly related to the genetic mutations (PINK1, BUB1B and COL16A1) or may be due to a supervening set of epigenetic alterations. The gene expression signature is dominated by alterations in cAMP/PKA signaling, and recent data has suggested a potential link between PINK1 and PKA signaling [32, 33]. Moreover, PINK1 is known to be induced by the PTEN pathway [34], which has previously been shown to be involved in melanoma progression and BRAF inhibitor resistance [35].

One limitation of our study is that identifying high confidence mutations in zebrafish tumors was surprisingly difficult, similar to what we have previously observed using exome approaches. We previously observed in an exome sequencing study a high rate of false positives with the available mutation callers at this time [8], but as we were using newer pipelines, we expected an improvement in performance we did not see. It is possible that we would need to sequence to a much higher depth to confidently call mutations in zebrafish tumors. It is also possible that utilizing new technologies with longer read lengths such as PacBio or Oxford nanopore would improve performance because we would be able to more confidently map mutations to the zebrafish reference genome.

## Conclusions

Complementing human cancer genomics with animals models can significantly augment our ability to “functionalize” the cancer genome. This can be done by testing candidate human mutations in fish or mouse models, but also by using these models to investigate underlying mutational mechanisms. The data highlighted here point the way forward for using zebrafish as a model for understanding mechanisms of genetic evolution in melanoma, but could easily be expanded to the wide range of other cancer models in the zebrafish. When coupled with the capacity for screens in the fish, one interesting future direction would then be to screen for factors that decrease the rate of genetic evolution, which would have direct relevance to human tumors.

## Methods

### Generation of transgenic zebrafish melanomas

The zebrafish melanomas were generated using the previously described miniCoopR system. Fish with the following genotype were incrossed: mitfa-BRAFV600E;p53-/-;mitfa-/-. These embryos were injected at the 1-cell stage with a DNA plasmid (containing the mitfa-MITF minigene along with an mitfa-EGFP sequence, surrounded by Tol2 transposon sites) along with Tol2 transposase mRNA.

### Isolation of the ZMEL1 and ZMELR1 melanoma lines

The ZMEL1 line was isolated as previously described. In brief, from the above transgenic group of animals, we isolated a fish with an obvious melanoma. This was then used to grow a stable zebrafish melanoma cell line which we refer to as ZMEL1. The ZMELR1 drug resistant line was derived by prolonged exposure of ZMEL1 to the BRAF inhibitor PLX4720 at a concentration of 10 μM. The drug was directly added to the cell culture media and the culture maintained for a period of ~4 months. Although most cells initially died after exposure to the drug, the culture eventually repopulated the culture flask and was then expanded for further studies. Due to better solubility, we switched the cells to the derivative drug PLX4032 (vemurafenib) at a concentration of 1 μM, which is essentially equivalent to the 10 μM concentration of PLX4720. The ZMELR1 line is continuously maintained in PLX4032 1 μM since removal of the drug tends to lead to growth slowdown, as has been previously described for other drug resistant melanoma lines.

### Whole-genome sequencing of the ZMEL1 line

Genomic DNA was isolated from normal muscle tissue from the original transgenic animal that gave rise to the melanoma used to make the ZMEL1 line. After a short period of culture, DNA was also then isolated from the ZMEL1 line. Approximately 1 μg of DNA for both the ZMEL1 and normal tissue was then subject to Illumina sequencing (HiSeq2000) using 100 bp PE sequencing to a depth of 39X.

Because of the generally high rate of single nucleotide polymorphisms in the zebrafish genome, we aligned the DNA reads to the zebrafish genome using the GSNAP aligner [14], which is generally more SNP tolerant than other aligners such as BWA. All reads were mapped to the Zv9 version of the zebrafish genome. After alignment, mutations were called by comparing the ZMEL1 DNA to the normal DNA from that fish, using the MuTect [15] and Shimmer [16] pipelines with default settings.

### MiSeq validation of ZMEL1 mutations

We divided the quality scores for both MuTect and Shimmer into quartiles. From each quartile, we then selected 48 candidate mutations for validation, resulting in 192 potential mutations from each caller, or 384 in total. Of these, 128 were mutations found in common between the two pipelines. We designed PCR primers to each of these loci, amplified them with high-fidelity polymerase (Kapa) and then pooled these products into a single MiSeq run.

We used three cutoffs for this analysis:

- SOMATIC (validated): > = 5% tumor allele frequency and < 2% normal allele frequency
- GERMLINE (not-validated): > = 5% tumor allele frequency and > = 2% normal allele frequency
- FAIL (not validated): <5% tumor allele frequency

### RNA-sequencing of the ZMELR1 and ZMEL1 line

Total RNA was isolated from both cell lines maintained in their standard culture conditions. Cells were 60–70% confluent at the time of RNA isolation. mRNA was prepared using the TruSEq RNA v2 kit, and 50 bp paired-end sequencing performed on the Illumina HiSeq2500, with ~20x10^6^ reads per sample.

Bioinformatics analysis was performed to systematically study differential expression and mutational changes in RNA-Seq profiles between control ZMEL1 cells (maintained in DMSO) and drug-resistant cell line ZMELR1 (maintained in PLX4032 1 μM). RNAseq samples were aligned to the Zebrafish genome (Version Zv9) using TopHat2 (v2.0.12) aligner [36]. With Ensembl zebrafish (Zv9, Ensembl release 79) as reference, aligned reads were then quantified for gene expression in terms of raw read counts and FPKM using HTSeq-counts [37] and Cufflinks [38], respectively. A count based differential expression was performed using the limma package in R [39]. Significantly differentially expressed genes were selected based on a cut-off of log fold change (logFC) >1 and False Discovery Rate (FDR) < 0.05.

For the mutation analysis, the RNASeq triplicates from control and ZMELR1 were merged to obtain one file per sample group, for high overall coverage and realigned to Zv9 using TopHat2. Mutation calls to obtain Single Nucleotide Variants (SNV) were then performed using Strelka comparing the control and ZMELR1 [40]. Based on Strelka’s thresholds, a predicted SNV was filtered in if the frequency of the variant allele in the ZMELR1 was significantly different from the reference allele in the control. The VCF output file from strelka, with the SNV candidates was the annotated with gene names and their predicted effects using SnpEff [41] with the Zv9 database.

### Ingenuity Pathway analysis of the ZMELR1 gene signature

From the differential expression analysis, the 852 zebrafish genes with FDR < 0.05 were selected for downstream analysis. In order to facilitate this, we mapped all of the zebrafish IDs to the human ortholog using the DIOPT tool (http://www.flyrnai.org/cgi-bin/DRSC_orthologs.pl), which resulted in 674 unique human IDs. These IDs, along with FDR and log2-fold change were then input into Ingenuity Pathway Analysis (http://www.ingenuity.com/products/ipa) using the default parameters (Direct and Indirect Relationships, no FDR or fold-change cutoff, all species consideredand all available data sources).

### Sanger validation of ZMELR1 mutations

From the Shimmer analysis, we identified 52 potential point mutations that were present in the ZMELR1 line but not in the ZMEL1 line itself. These were stratified based on variant allele frequency, ranging from 100% down to 3.4%. We chose to validate 28 genes with a range of variant allele fractions. To do this, we isolated genomic DNA from both the ZMEL1 and ZMELR1 line, and then designed PCR primers around the putative mutation, similar to what we did for the validation of the ZMEL1 mutations. These loci were amplified with high fidelity polymerase, and then individually sequenced using Sanger sequencing.

## Additional files

Additional file 1: Table S1. ZMEL1 whole-genome sequencing. This file contains all of the mutations called by MuTect and Shimmer, along with their associated quality scores and overlapping mutations. (XLSX 6276 kb)
Additional file 2: Table S2. ZMEL1 validation primers for MiSeq run. This file contains all of the PCR primer sequences used to validate the subset of 384 called mutations that were validated in the ZMEL1 line via MiSeq. (XLSX 58 kb)
Additional file 3: Table S3. ZMEL1 MiSeq validation calls. This file contains all of the mutation calls for the MiSeq validation run of the ZMEL1 line. (XLSX 178 kb)
Additional file 4: Table S4. ZMELR1 vs. ZMEL1 mRNA differential expression. This file contains the differentially expressed genes from RNA-seq of the ZMEL1 line vs. the ZMELR1 line. (XLSX 3569 kb)
Additional file 5: Table S5. ZMELR1 vs. ZMEL1 candidate mutations. This file contains the candidate mutations called from the RNA-seq in the ZMELR1 line compared to the ZMEL1 line. (XLSX 21 kb)

## Abbreviations

UV: Ultraviolet light
